# SCOPA and META-SCOPA: software for the analysis and aggregation of genome-wide association studies of multiple correlated phenotypes

**DOI:** 10.1186/s12859-016-1437-3

**Published:** 2017-01-11

**Authors:** Reedik Mägi, Yury V. Suleimanov, Geraldine M. Clarke, Marika Kaakinen, Krista Fischer, Inga Prokopenko, Andrew P. Morris

**Affiliations:** 1 Estonian Genome Center, University of Tartu, Tartu, Estonia; 2 Computation-based Science and Technology Research Center, Cyprus Institute, Nicosia, Cyprus; 3 Department of Chemical Engineering, Massachusetts Institute of Technology, Cambridge, MA USA; 4 Wellcome Trust Centre for Human Genetics, University of Oxford, Oxford, UK; 5 Genomics of Common Disease, Imperial College, London, UK; 6 Department of Biostatistics, University of Liverpool, Liverpool, UK

**Keywords:** Genome-wide association study, Multivariate analysis, Reverse regression, Correlation, Multiple phenotypes, Meta-analysis

## Abstract

**Background:**

Genome-wide association studies (GWAS) of single nucleotide polymorphisms (SNPs) have been successful in identifying loci contributing genetic effects to a wide range of complex human diseases and quantitative traits. The traditional approach to GWAS analysis is to consider each phenotype separately, despite the fact that many diseases and quantitative traits are correlated with each other, and often measured in the same sample of individuals. Multivariate analyses of correlated phenotypes have been demonstrated, by simulation, to increase power to detect association with SNPs, and thus may enable improved detection of novel loci contributing to diseases and quantitative traits.

**Results:**

We have developed the SCOPA software to enable GWAS analysis of multiple correlated phenotypes. The software implements “reverse regression” methodology, which treats the genotype of an individual at a SNP as the outcome and the phenotypes as predictors in a general linear model. SCOPA can be applied to quantitative traits and categorical phenotypes, and can accommodate imputed genotypes under a dosage model. The accompanying META-SCOPA software enables meta-analysis of association summary statistics from SCOPA across GWAS. Application of SCOPA to two GWAS of high-and low-density lipoprotein cholesterol, triglycerides and body mass index, and subsequent meta-analysis with META-SCOPA, highlighted stronger association signals than univariate phenotype analysis at established lipid and obesity loci. The META-SCOPA meta-analysis also revealed a novel signal of association at genome-wide significance for triglycerides mapping to *GPC5* (lead SNP rs71427535, *p* = 1.1x10^−8^), which has not been reported in previous large-scale GWAS of lipid traits.

**Conclusions:**

The SCOPA and META-SCOPA software enable discovery and dissection of multiple phenotype association signals through implementation of a powerful reverse regression approach.

## Background

In the past decade, genome-wide association studies (GWAS) of single nucleotide polymorphisms (SNPs) have proven to be successful in identifying loci contributing genetic effects to a wide range of complex human traits, including susceptibility to diseases [1]. Interestingly, many of these loci harbour SNPs that are associated with multiple phenotypes, some of which are correlated with each other (such as serum lipid concentrations [2]) or share underlying pathophysiology (such as chronic inflammatory diseases [3]), whilst others are epidemiologically unrelated.

The observation of multiple phenotype association at the same locus can occur as a result of pleiotropy [4]. Biological pleiotropy describes the scenario in which SNPs in the same gene are directly causal for multiple phenotypes. Biological pleiotropy can be considered: (i) at the “allelic level”, where the causal variant is the same for all phenotypes; (ii) due to “co-localisation”, for which the causal variants are not the same for all phenotypes, but are correlated with each other (i.e. in linkage disequilibrium); or (iii) at the “genic level”, where the causal variants are not the same for all phenotypes, and are uncorrelated with each other. Mediated pleiotropy occurs when a SNP is directly causal for one phenotype, which is in turn correlated, epidemiologically, with others. Spurious pleiotropy refers to multi-phenotype associations that do not reflect shared underlying genetic pathways, and can occur when causal variants act through different genes at the same locus, as a result of confounding that is not adequately accounted for in the analysis, or due to misclassification or ascertainment bias in disease cases.

The traditional approach to the analysis of GWAS is to consider each phenotype separately (i.e. univariate), despite the fact that many diseases and quantitative traits are correlated with each other, and often measured in the same sample of individuals. However, under these circumstances, there may be increased power to detect novel loci associated with multiple phenotypes through multivariate analyses [5]. A wide range of methods have been proposed, including multivariate analysis of variance [6], dimension reduction [7, 8], generalised estimating equations [9], Bayesian networks [10], and non-parametric approaches [11]. The most suitable approach will often depend on study design because, for example, methods may be restricted to the analysis of quantitative traits, or cannot accommodate covariates.

One of the most flexible multivariate methods for multiple phenotype analysis uses “reverse regression” techniques. With this approach, phenotypes are used as predictors of genotype at a SNP in an ordinal regression model [12]. Unlike multivariate analysis of variance, as implemented in the MAGWAS software [6], reverse regression has the advantage that it can simultaneously incorporate both quantitative traits and categorical phenotypes in the same model. Simulations have also demonstrated that this approach has a dramatic increase in power over univariate analyses in many scenarios, whilst controlling false positive error rates [12]. Reverse regression has the disadvantage, however, that model parameter estimates cannot be directly interpreted in terms of the effect of a SNP on each phenotype. The reverse regression approach has been previously implemented in the MultiPhen package: https://cran.r-project.org/web/packages/MultiPhen/index.html.

Here we implement a reverse regression model for multiple correlated phenotypes in SCOPA (Software for COrrelated Phenotype Analysis) that has a number of key advantages over MultiPhen. First, the software can accommodate directly typed and imputed SNPs (under an additive dosage model), appropriately accounting for uncertainty in the imputation in the downstream association analysis. Second, dissection of multivariate association signals is achieved through model selection to determine which phenotypes are jointly associated with the SNP. Third, SCOPA association summary statistics can also be aggregated across GWAS through fixed-effects meta-analysis, implemented in META-SCOPA, enabling application of reverse regression in large-scale international consortia efforts where individual-level genotype are phenotype data cannot be shared between studies.

To demonstrate the power and utility of this approach, we apply the software to two GWAS of high— and low-density lipoprotein (HDL and LDL) cholesterol, triglycerides (TG) and body mass index (BMI), and evaluate association signals in established lipid and obesity loci.

## Implementation

### Reverse regression model of multiple correlated phenotypes

Consider a sample of unrelated individuals with *J* phenotypes denoted by ***y***_1_, ***y***_2_, …, ***y***_*J*_. At a SNP, we denote the genotype of the *i*th individual by *G*_*i*_, coded under an additive model in the number of minor alleles (dosage after imputation). Under linear reverse regression, we model the genotype as a function of the observed phenotypes, such that

1 $$ {G}_i=\alpha +{\displaystyle {\sum}_j{\beta}_j{y}_{ij}+{\epsilon}_i}. $$

In this expression, *β*_*j*_ denotes the effect of the *j*th phenotype on genotype at the SNP, and *ϵ*_*i*_ ~ *N*(0, *σ* ^2^), where *σ* ^2^ is the residual variance. A joint test of association of the SNP with the phenotypes, with *J* degrees of freedom is constructed by comparing the maximised log-likelihood of the unconstrained model (1), with that obtained under the null model, for which **β** = **0**. The maximum likelihood estimate, ${\widehat{\beta}}_j$, of the effect of the *j*th phenotype is adjusted for all other traits included in the reverse regression model, and thus implicitly accounts for the correlation between them.

It is important to account for potential confounding, for example arising as a result of population structure. We therefore recommend that phenotypes are replaced by residuals after adjustment for “general” confounders, such as age, sex and principal components to account for population structure, as covariates in a generalised linear modelling framework. However, where a potential confounder might share genetic effects with the phenotypes under investigation, such as body-mass index in the analysis of waist-hip ratio, we would recommend including this as an additional variable in the reverse regression model.

### Dissection of multiple phenotype association signals

For SNPs attaining genome-wide significant evidence of association (*p* <5×10^−8^) with the phenotypes, it may be of interest to further dissect the signal through model selection. We obtain a maximised log-likelihood of the model (1) for each possible subset of phenotypes (so that *β*_*j*_ = 0 if the *j*th phenotype is excluded from the model). We then determine the “best” subset of phenotypes associated with the SNP as the model with minimum Bayesian information criterion (BIC).

### Meta-analysis

Consider *K* GWAS of the same set of correlated phenotypes. At a SNP, we denote the maximum likelihood estimates of the effect of the phenotypes from the *k*th GWAS by ${\widehat{\boldsymbol{\upbeta}}}_k$, with corresponding variance-covariance matrix **V**_*k*_. Association summary statistics are then aggregated across studies using the method for the synthesis of regression slopes [13]. The BIC for each model for a SNP can also be aggregated across GWAS to enable dissection of the association signal after meta-analysis.

### Genomic control

To correct for residual population structure within and between GWAS, which is not accounted for in study-level association analyses, we calculate the genomic control inflation factor, *λ*, on the basis of *J* degrees of freedom, one for each phenotype [14]. The inflation factor is calculated at the study level (denoted *λ*_*k*_ for the *k*th GWAS) and after meta-analysis (denoted *λ*_MA_), enabling “double” genomic control correction. Elements of the variance-covariance matrix of the *k*th study, **V**_***k***_, are inflated by *λ*_*k*_, unless *λ*_*k*_ <1. Similarly, elements of the variance-covariance matrix after meta-analysis are inflated by *λ*_MA_, unless *λ*_MA_ <1.

### SCOPA and META-SCOPA

Genome-wide study-level multiple phenotype analysis, including dissection of association signals, has been implemented in SCOPA. The software requires specification of input genotype and sample files, and a list of phenotypes to be included in the analysis. SCOPA includes options to enable filtering on the basis of imputation quality (info score) [15], to output the variance-covariance matrix and phenotype effects (with standard errors) for each SNP, and to investigate association with all possible subsets of phenotypes using BIC.

Genome-wide meta-analysis has then been implemented in META-SCOPA. The software requires specification of a list of SCOPA output files representing studies to be included in the meta-analysis. META-SCOPA includes options to enable genomic control correction (at the study level and/or after meta-analysis), and filtering of SNPs on the basis of minor allele frequency (MAF) and imputation quality.

### Required file formats

SCOPA requires genotype and phenotype data in GEN/SAMPLE file format utilised by IMPUTE and SNPTEST [15–17]. This format accommodates imputed genotype data in the GEN file and multiple phenotypes in the SAMPLE file. Full details of the file formats can be found at: http://www.stats.ox.ac.uk/~marchini/software/gwas/file_format.html. Conversion to GEN/SAMPLE files from other formats for genotype/phenotype data can be performed using GTOOL: http://www.well.ox.ac.uk/~cfreeman/software/gwas/gtool.html.

## Results and discussion

We considered two GWAS of LDL cholesterol, HDL cholesterol, TG and BMI from the Estonian Biobank at the Estonian Genome Center, University of Tartu [18]. Individuals from the EGCUT-OMNI GWAS were genotyped with the Illumina HumanOmniExpress BeadChip, whilst those from the EGCUT-370 GWAS were genotyped with the Illumina HumanCNV370 BeadChip. In both studies, individuals were excluded on the basis of call rate <95%, gender discordance with X chromosome genotypes, and excess heterozygosity (>3 standard deviations). After quality control 609 and 832 individuals, respectively, were retained in EGCUT-OMNI and EGCUT-370. SNPs were excluded on the basis of call rate <95%, extreme deviation from Hardy-Weinberg equilibrium (*p* <10^−6^), and MAF <1%. Principal components were derived from a genetic related matrix in each study to account for population structure in downstream association analyses [19]. The genotype scaffold of individuals and SNPs passing quality control was pre-phased, separately in each study, using SHAPEIT [20]. The phased scaffold was then imputed up to the 1000 Genomes Project Consortium reference panel (all ancestries, June 2011 release) [21], separately in each study, using IMPUTEv2 [15, 16]. SNPs with MAF <1% and imputation quality info score <0.4 were excluded from downstream association analyses.

In both studies, HDL cholesterol, LDL cholesterol and TG were measured from serum extracted from whole blood. Lipid measurements deviating more than 5 standard deviations from the mean were set to missing. Individuals were excluded if they received lipid-lowering medication at sample collection. The four phenotypes were adjusted for age, age^2^ [2] and four principal components to account for population structure. Residuals were calculated separately for men and women, and inverse standard normal transformed by the inverse standard normal function.

We applied SCOPA to the four phenotypes in each GWAS, and aggregated association summary statistics across studies using META-SCOPA. There was no evidence for residual population structure within and between GWAS that was not accounted for in the association analysis: *λ*_OMNI_ = 1.001 and *λ*_370_ = 0.999 for EGCUT-OMNI and EGCUT-370, respectively, at the study level, and *λ*_MA_ = 1.003 after meta-analysis.

**Fig. 1:**
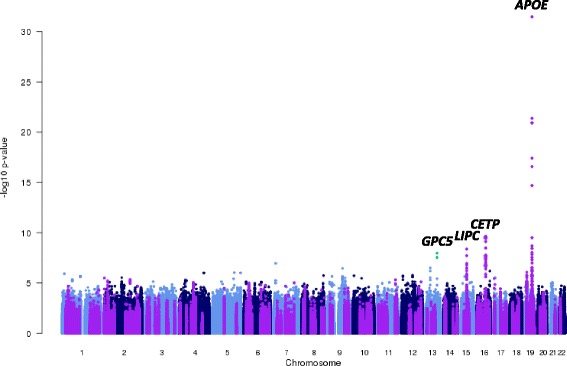
Manhattan plot of META-SCOPA meta-analysis of GWAS of lipid traits and BMI in 1,441 individuals from the Estonian Genome Center, University of Tartu. Each point represents a SNP passing quality control, plotted according to their genomic position (NCBI build GRCh37, UCSC hg19 assembly) on the x-axis and their *p*-value for multiple phenotype association (on -log_10_ scale) on the y-axis. Previously reported loci for lipid traits and BMI are highlighted in *purple*. Names of loci attaining genome-wide significance (*p* <5x10^−8^) are reported as the nearest gene to the lead SNP, unless a better biological candidate maps nearby. SNPs attaining genome-wide significant, but not mapping to previously reported loci for lipid traits or BMI, are highlighted in *green*

**Fig. 2:**
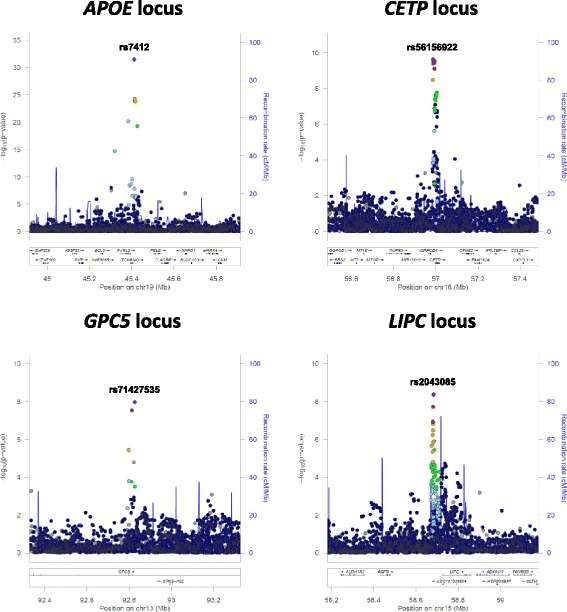
Signal plots for loci attaining genome-wide significance (*p* <5x10^−8^) from META-SCOPA meta-analysis of GWAS of lipid traits and BMI in 1,441 individuals from the Estonian Genome Center, University of Tartu. Each point represents a SNP passing quality control in the association analysis, plotted with their *p*-value (on a -log_10_ scale) as a function of genomic position (NCBI build GRCh37, UCSC hg19 assembly). In each plot, the lead SNP is represented by the purple symbol. The colour coding of all other variants indicates linkage disequilibrium with the lead SNP in European ancestry haplotypes from the 1000 Genomes Project reference panel: *red r* ^2^ ≥0.8; *gold* 0.6 ≤ *r* ^2^ <0.8; *green* 0.4 ≤ *r* ^2^ <0.6; *cyan* 0.2 ≤ *r* ^2^ <0.4; *blue r* ^2^ <0.2; grey *r* ^2^ unknown. Recombination rates are estimated from Phase II HapMap and gene annotations are taken from the University of California Santa Cruz genome browser

Our META-SCOPA analysis revealed four loci attaining genome-wide significant evidence of association (*p* <5×10^−8^) with lipids and BMI (Figs. 1 and 2, Table 1), mapping to/near: *APOE* (rs7412, *p* = 3.4×10^−32^); *CETP* (rs56156922, *p* = 2.4×10^−10^); *GPC5* (rs71427535, *p* = 1.1×10^−8^); and *LIPC* (rs2043085, *p* = 1.9×10^−8^). For comparison, we also performed univariate tests of association in SCOPA for each phenotype, separately, within each GWAS, and aggregated summary statistics across studies through fixed-effects meta-analysis (inverse-variance weighting of effect sizes) using GWAMA [22]. After correcting for testing of four traits with Sidak’s adjustment, the signals of association at each locus from SCOPA were always stronger than observed in univariate analysis (Table 2).

**Table 1:** Loci attaining genome-wide significance (*p* <5×10^−8^) in META-SCOPA meta-analysis of GWAS of lipid traits and BMI in 1,441 individuals from the Estonian Genome Center, University of Tartu

Locus	Lead SNP	Chr	Position^a^ (bp)	Alleles		EAF	META-SCOPA				
				Effect	Other		BMI effect (SE)	HDL effect (SE)	LDL effect (SE)	TG effect (SE)	*p*-value
*APOE*	rs7412	19	45,412,079	T	C	0.102	−0.017 (0.011)	−0.046 (0.011)	0.129 (0.011)	−0.078 (0.012)	3.4×10^−32^
*CETP*	rs56156922	16	56,987,369	C	T	0.308	−0.026 (0.017)	−0.119 (0.018)	0.046 (0.017)	−0.024 (0.019)	2.4×10^−10^
*GPC5*	rs71427535	13	92,826,439	C	T	0.108	0.007 (0.011)	0.014 (0.012)	0.024 (0.011)	−0.065 (0.012)	1.1×10^−8^
*LIPC*	rs2043085	15	58,680,954	T	C	0.343	−0.022 (0.019)	−0.124 (0.020)	0.013 (0.019)	−0.070 (0.020)	1.9×10^−8^

Chr: chromosome. SE: standard error. EAF: effect allele frequency

^a^Position reported for NCBI build GRCh37 (UCSC hg19 assembly)

**Table 2:** Univariate GWAS meta-analysis of lipid traits and BMI at lead SNPs in 1,441 individuals from the Estonian Genome Center, University of Tartu

Locus	Lead SNP	Chr	Position^a^ (bp)	Alleles		BMI		HDL		LDL		TG	
				Effect	Other	Effect (SE)	*p*-value	Effect (SE)	*p*-value	Effect (SE)	*p*-value	Effect (SE)	*p*-value
*APOE*	rs7412	19	45,412,079	T	C	−0.018 (0.011)	0.41	−0.015 (0.011)	0.55	0.107 (0.011)	1.9×10^−23^	−0.025 (0.011)	0.093
*CETP*	rs56156922	16	56,987,369	C	T	−0.007 (0.017)	0.99	−0.105 (0.016)	6.1×10^−10^	0.040 (0.017)	0.062	0.032 (0.017)	0.19
*GPC5*	rs71427535	13	92,826,439	C	T	−0.003 (0.011)	1.0	0.038 (0.011)	0.0012	0.006 (0.011)	0.96	−0.063 (0.011)	1.3×10^−8^
*LIPC*	rs2043085	15	58,680,954	T	C	−0.009 (0.019)	0.98	−0.093 (0.018)	6.5×10^−7^	−0.005 (0.018)	1.0	−0.019 (0.018)	0.74

Chr: chromosome. SE: standard error

^a^Position reported for NCBI build GRCh37 (UCSC hg19 assembly)

The lead SNP at the *APOE* locus, rs7412, has been previously reported, at genome-wide significance, in univariate GWAS meta-analysis of lipid traits [23], where the primary signal is with LDL cholesterol, but also with strong associations with HDL cholesterol and TG. This lead SNP is one of two tags that define *APOE* ε2/ε3/ε4 alleles [23]. Genetic variation at *CETP* and *LIPC* has also been previously implicated in univariate GWAS meta-analysis of lipid traits, where the primary associations are with HDL cholesterol [2, 23, 24]. Our lead SNPs at these loci are in strong linkage disequilibrium with those previously reported [23] (*r* ^2^ = 0.971 between rs56156922 and rs17231506 at *CETP*; *r* ^2^ = 0.849 between rs2043085 and rs261291 at *LIPC*), suggesting that they represent the same underlying association signals. The *APOE* locus has also formerly been associated with BMI, at genome-wide significance, in univariate GWAS meta-analysis [25, 26], although the lead SNP from SCOPA is independent of that previously reported (*r* ^2^ = 0.013 between rs7412 and rs2075650), suggesting that this signal is distinct from that identified for LDL cholesterol.

Genetic variation at the *GPC5* locus has not been previously associated with lipid traits or BMI at genome-wide significance. The lead SNP, rs71427535, maps to an intron of *GPC5* (Glypican 5), a gene that plays a role in the control of cell division and growth regulation. The gene is involved in retinoid and carbohydrate metabolic processes, making it a highly plausible candidate gene for lipid metabolism, although further replication of the association signal in additional studies is required.

We dissected multiple phenotype association signals for the lead SNPs at the four loci attaining genome-wide significance after meta-analysis. We determined the best subset of phenotypes according to the BIC across studies, which represents a trade off in overall model fit with the number of parameters required (Table 3). At *CETP* and *LIPC*, the phenotype subset with minimum BIC for the lead SNPs included only HDL cholesterol. This model is consistent with previous reports [2, 23] that the primary associations at these loci are with HDL cholesterol, and that GWAS signals for other lipids at these lead SNPs are likely driven through mediated pleiotropy. At *GPC5*, the phenotype subset with minimum BIC for the lead SNP included only TG, suggesting that the primary association signal at this locus is driven by this specific serum lipid trait. Finally, at *APOE*, the phenotype subset with minimum BIC for the lead SNP included HDL cholesterol, LDL cholesterol and TG. Previous reports have highlighted association signals with multiple lipid traits at this locus [2, 23, 24]. Our analyses suggest that the multiple phenotype associations are not entirely driven by correlation between lipids and mediation through LDL cholesterol, but highlight biological pleiotropy as a possible driving mechanism. However, further dissection of this locus in larger samples is required to confirm this assertion, and causal relationships between these phenotypes cannot be established without more detailed Mendelian randomisation studies, for example.

**Table 3:** Dissection of multiple phenotype association signals for lead SNPs from META-SCOPA meta-analysis of GWAS of lipid traits and BMI in 1,441 individuals from the Estonian Genome Center, University of Tartu

Model	Difference in BIC from null model			
	*APOE*: rs7412	*CETP*: rs56156922	*GPC5*: rs71427535	*LIPC*: rs2043085
BMI	10.29	12.64	12.99	12.73
HDL	10.85	−28.53	0.06	−14.75
LDL	−86.12	7.26	8.86	10.31
TG	7.18	9.36	−21.69	11.95
BMI + HDL	20.75	−17.41	13.01	−3.44
BMI + LDL	−76.28	19.96	21.91	23.01
BMI + TG	17.67	21.95	−9.23	24.61
HDL + LDL	−75.72	−21.24	9.37	−3.94
HDL + TG	14.04	−15.75	−10.66	−13.61
LDL + TG	−105.91	18.66	−17.68	21.70
BMI + HDL + LDL	−66.14	−10.18	22.26	7.25
BMI + HDL + TG	24.27	−4.57	1.50	−1.62
BMI + LDL + TG	−95.35	31.31	−5.21	34.30
HDL + LDL + TG	−108.01	−10.02	−5.82	−4.07
BMI + HDL + LDL + TG	−97.59	1.25	6.33	7.94

## Conclusions

The SCOPA and META-SCOPA software enable discovery and dissection of multiple phenotype association signals through implementation of a powerful reverse regression approach. Application of the software to two GWAS of HDL and LDL cholesterol, TG and BMI highlighted stronger association signals than univariate phenotype analysis at established lipid and obesity loci. The meta-analysis also revealed a novel signal of association for triglycerides mapping to *GPC5* (lead SNP rs71427535, *p* = 1.1×10^−8^), which has not been reported in previous GWAS of lipid traits. Dissection of the *APOE* locus highlighted associations with LDL and HDL cholesterol and TG, and suggested biological pleiotropy as a likely driving mechanism for this multiple lipid signal.

## Availability and requirements

Project name: SCOPA.

Availability: the SCOPA and META-SCOPA software, documentation and tutorial can be found at: http://www.geenivaramu.ee/en/tools/scopa.

Operating system(s): Linux.

Programming language: C++ (including files from the ALGLIB project for statistical analysis and the TCLAP project for command line argument parsing).

Any restrictions on use by academics: none.

## Abbreviations

BIC: Bayesian information criterion
BMI: Body mass index
GWAS: Genome-wide association study
HDL: High-density lipoprotein
LDL: Low-density lipoprotein
MAF: Minor allele frequency
SNP: Single nucleotide polymorphism
TG: Triglycerides
